# Incidence rate, risk factors, and bacterial causes of clinical mastitis on dairy farms in Hawassa City, southern Ethiopia

**DOI:** 10.1038/s41598-023-37328-1

**Published:** 2023-07-06

**Authors:** Rahmeto Abebe, Alemayehu Markos, Mesele Abera, Berhanu Mekbib

**Affiliations:** 1 Faculty of Veterinary Medicine, Hawassa University, P.O.Box 05, Hawassa, Ethiopia; 2 Damboya District Agricultural Development Office, KT Zone, SNNPRS Damboya, Ethiopia

**Keywords:** Microbiology, Diseases, Risk factors

## Abstract

Mastitis is the most common disease of dairy cattle worldwide, causing economic losses due to reduced yield and poor quality of milk. It is of particular concern in Ethiopia, where effective prevention and control practices are lacking. The objective of the present prospective longitudinal study was to estimate the incidence rate of clinical mastitis (CM), identify the risk factors, isolate the bacterial agents, and determine the risk of recurrence. For this purpose, a total of 217 lactating cows were followed up every two weeks from calving to drying off or the end of the study period. Of these, 79 (36.41%) developed CM, of which 23% had recurrent infections in the same or a different quarter. The overall incidence rate of CM was 83.72 (95% CI: 63.2–98.2) cases per 100 cow-years at risk. In the multivariable Cox regression model, the risk of CM was found to be significantly higher in multiparous cows (HR = 1.96; p = 0.03), in cows with a history of mastitis (HR = 2.04; p = 0.030), in cows that had severely keratinized teat end condition (HR = 7.72; p < 0.001) and in cows kept in poorly cleaned barns (HR = 1.89; p = 0.007). The pathogenic bacteria isolated from mastitis-positive cows were *Staphylococcus aureus* (28.1%), *E. coli* (21.1%), *Bacillus* spp. (14%), *Streptococcus* spp. (14%), coagulase-negative staphylococci (12.3%), non aureus staphylococci (5.3%), *Enterobacter* spp. (3.5%), *Klebsiella* spp. (1.8%), *Corynebacterium* spp. (1.8%), and *Proteus* spp. (1.8%). The high incidence rate of CM in the present study shows that the disease spreads rapidly and can be responsible for a significant economic loss to milk producers in the study area. Therefore, raising awareness among dairy farmers, early detection and treatment of cases, post-milking teat disinfection, improvement of the hygienic status of cows and barns, use of dry cow therapy, and culling of chronic cases are recommended as viable measures to prevent and control clinical mastitis in the study area.

## Introduction

Ethiopia holds great potential for dairy development due to its large cattle population of nearly 60 million^1^. In addition, the country has a favorable climate for improved high-yielding animal breeds. Currently, there is a rise in dairy farms in urban and peri-urban areas due to an increased demand for dairy products resulting from a combination of factors such as population growth, urbanization, and an increasing understanding of the nutritional benefits of milk. Although the country has a large herd of cattle, milk production is still well below expected levels due to various factors, including udder health problems. One of the major udder health problems affecting milk production and quality is mastitis^2^.

Mastitis is an inflammation of the parenchyma of the mammary gland caused by numerous and diverse organisms, mainly bacteria. Depending on the severity and course of the inflammation, mastitis can be clinical or subclinical^3^. Clinical mastitis (CM) is obvious and easily recognized by visible abnormalities, such as red and swollen udders and fever in dairy cows. The cow’s milk appears watery with the presence of flakes and clots. Unlike CM, subclinical mastitis shows no visible abnormalities in the udder or milk, but milk production decreases with an increase in the somatic cell count^4^. CM is an economically important disease affecting the dairy industry worldwide. Losses due to CM include severe milk losses, reduced milk quality, increased treatment costs, veterinary costs, extra labor, and an increased likelihood of death and culling of the affected dairy cows^5^. In addition to these economic aspects, CM is a recurring event that leads to a higher susceptibility to further episodes of mastitis in the same or the next lactation^6,7^.

Studies in different countries indicate that CM is a multifactorial disease resulting from exposure to pathogens, the presence of animal and herd-level risk factors, and interactions between these factors^8^. Currently, known risk factors for CM come mainly from studies in Europe and North America. Accordingly, the herd-specific risk factors for CM include a lack of dry cow therapy, milking machines with insufficient suction pressure, and shared udder preparation tissues^9^. The cow-specific risk factors reported were parity, the month of lactation, somatic cell count in the previous lactation, and history of CM^10,11^. Environmental factors such as the season of the year have also been mentioned as risk factors for CM^12^. As the farm management practices in Ethiopia differ from those in Europe and North America, the identification of area-specific risk factors is of paramount importance to design an effective CM prevention and control program that is specific to the local situation.

Over the years, several researchers have studied the prevalence, risk factors, and main causes of mastitis in dairy cattle in different parts of Ethiopia. Studies have shown that the prevalence of mastitis, both clinical and subclinical ranges from 17.1% to 71%. Several of these studies have demonstrated the presence of a range of mastitis-causing bacteria, indicating *Staphylococcus aureus* and *Streptococcus agalactiae* as the dominant pathogenic species^13–17^. Studies have also shown that mastitis is the first most important problem on large farms and the second most important next to reproductive disorders on small farms^18^.

However, most mastitis studies available in Ethiopia have been cross-sectional, examining the prevalence, risk factors, and bacterial etiologies of subclinical mastitis at a single time point, lactation stage, and season. Due to the design used, the previous studies did not show the incidence rate of new cases per farm per year and the speed at which the disease spreads between cows in a herd. Furthermore, information on the risk factors and bacterial causes of CM and the risk of recurrence of CM in the same lactation in Ethiopia is lacking. Prospective longitudinal studies provide estimates of disease incidence rates and are more suited to establish cause-and-effect relationships than cross-sectional studies. The current study was designed to fill the available information gap by following lactating cows throughout lactation for the occurrence of CM. Therefore, the study aimed to determine the incidence rate of CM, isolate all pathogenic bacteria likely to cause the disease, identify associated risk factors, and determine the risk of recurrence of CM in the dairy farms of Hawassa City, which is one of the areas with high potential for milk production in southern Ethiopia.

## Materials and methods

### Study area

This study was conducted on selected dairy farms located in Hawassa City between October 2018 and August 2019. Hawassa is the capital of Sidama Regional State and is situated on the shores of Lake Hawassa in the Great Rift Valley and is 275 km south of Addis Ababa, the capital, along the Addis Ababa—Moyale highway. The city lies at an altitude of 1708 m above sea level and is located at 7^0^ 3' north latitude and 38^0^ 29' east longitude (Supplementary file 1). The city’s annual average rainfall ranges from 800 to 1000 mm, while the average annual temperature varies from 20.1 to 25 °C^19^.

### Study population and farm management

The study population included all lactating crossbred cows found on the selected dairy farms. In terms of breed, most of the cows (88%) were Holstein–Friesian local crosses and a few Jersey local crosses (12%). According to the Hawassa City Livestock and Fisheries Department (2019), there are 109 registered dairy farms in the city with herd sizes ranging from 20 to 107 heads. All cows on the farms were kept on the zero-grazing system (intensive), except one dairy farm, where the cows were kept on a semi-intensive basis. In the latter case, the cows were allowed to graze on the farm’s premises for approximately 6 h a day and were sometimes supplemented with some amounts of concentrates. The animals on this farm feed on a straw of wheat and teff most of the time. On all other farms, the feeding consists of roughages and concentrates. The common types of concentrates offered to dairy cows include wheat bran, noug seed cake, wheat middling, linseed cake, bean hulls, and others. The cows received concentrated feed at least twice a day, but the amount may vary from farm to farm and within a farm. Common roughage forages used on the farms included teff straw, green grasses, hay, and kidney bean straw (most common on all farms). In all farms, the feeding trough was separate for each animal except in one dairy farm, in which animals had a common feeding trough depending on their age.

All farms use a tie-stall system except one dairy farm which uses a group housing system. In group stalls, the animals were tied up only during milking. All tie stalls had a roof and were enclosed with a sidewall, except for one farm which had no sidewall. Only two of the dairy farms had a calving pen, which was dry and had a concrete floor. On all farms, the cows were milked by hand twice a day in the morning and in the evening. Only two dairy farms had a paddock for cows. The paddock has a roof on one of the farms but not on the other.

### Study design and sampling method

A prospective, longitudinal study design was used to address the objectives of the current study. To this end, individual sample units (cows) were identified and monitored regularly every two weeks throughout the study period or until the end of lactation to detect the emergence of new CM cases in the herds. Since there are large differences in herd size among the dairy farms in the study area we specifically selected only farms with 10 or more cows to increase the likelihood of finding more than one lactating cow for follow-up. Of the 109 registered dairy farms in Hawassa City, 22 farms had 10 or more cows. A letter of cooperation was sent to these farms of which 19 agreed to participate in the study. Therefore, the number of farms included in our study was 19. The selected farms represent 17.4% of the dairy farms in the city. At the start of the study, all lactating cows with lactation durations of no more than three weeks after calving were recruited for the study. In addition, cows that calved during the study were also included. Accordingly, the total number of cows monitored for the incidence rate of CM during the study period was 217.

### Data collection

#### Questionnaire survey

A semi-structured questionnaire was designed according to Dohoo et al.^20^ and administered to the owners or managers of the selected dairy farms through face-to-face interviews to collect information on the dairy farm management practices and cow-level data, which were used to assess risk factors for CM. Farm-level data collected included cleaning of stalls, ventilation, use of bedding materials in stalls, feeding system, herd size, dry cow therapy, preparation of udder and teats before milking (whole udder or teats only), use of towels to dry udder and teats, teat disinfection before or after milking, milking a mastitic cow or quarter last, culling chronically affected cows, whether CM is a problem on the farm, treatment of CM, action on the milk during antibiotic treatment, the person responsible for running the farm (owner or employed manager), the level of training of the manager and the presence of employed veterinarian on the farm. Animal-related data collected included age, breed, parity number, history of mastitis in the previous lactation, cow soiling (udder and leg hygiene score), teat end morphology (pointed, round, or flat), teat end score, calving date, and average milk yield per day.

#### Monitoring of cows

At the 19 selected farms, lactating cows recruited for the study were monitored every two weeks until drying off or the end of the study period. Thus, each farm was visited a maximum of 21 times during the study. At each visit, the investigator inquired about the occurrence of CM in the study cows within the previous two weeks, followed by a physical examination of the udder and milk changes through visualization, palpation, and milking of healthy and suspected cows. The information collected from each visit was recorded in the data collection sheet that was prepared separately for each farm. Once a cow developed CM in one or more quarters, she was withdrawn from further monitoring but followed up for recovery of the affected quarter or recurrence of CM in the same or another quarter. If cows were lost to follow-up, the date and reason were recorded.

#### Microbiological analysis of milk samples

Approximately 10 ml milk sample was aseptically collected by a trained graduate student (the second author) from 57 selected mastitis-positive cows into a sterile, narrow-necked bottle. The samples were labeled, placed in a cooler with ice bags, and shipped to the Hawassa University Faculty of Veterinary Medicine Microbiology Laboratory within 3 h. Up on arrival, the samples were cultured on the same day of collection. However, if this was not possible, the samples were stored at 4 °C for a maximum of 24 h. Bacterial isolation and identification was performed according to National Mastitis Council (NMC) guidelines^21^ using Sheep Blood Agar (SBA) and MacConkey Agar (MAC). Briefly, a loop full of milk was streaked onto SBA and MCA plates and incubated at 37 °C for 24 to 72 h. Gram-positive bacteria were identified based on growth characteristics on SBA, then mannitol salt agar (MSA) followed by the catalase test, coagulase test, and gram staining. Gram-negative bacteria were identified by oxidase test as well as by growth features on MAC and eosin methylene blue agar (EMB). If the colonies on SBA had β hemolysis, a positive catalase test, yellow color formation on MSA, and a positive result in the tube coagulase test, then they were considered *S. aureus*. Those with no hemolysis on SBA, a positive catalase test, yellow or pink color formation in MSA, and a negative coagulase test were considered non-aureus staphylococci (NAS). *Staphylococcus* spp. that were coagulase-negative and MSA-negative were classified as coagulase-negative staphylococci (CNS). Very small colonies with/without β hemolysis on SBA, no growth on MAC, a negative catalase test and violet-colored short- or long-chain cocci on Gram stain were considered streptococci. Large greyish colonies with β hemolysis on SBA but no growth on MAC with large violet bacilli in Gram staining were considered *Bacillus* spp. Colonies that were not hemolytic but whitish, small, dry, and grew heavily in the initial inoculating area on SBA, had no growth on MAC, Gram-positive short rods or coccobacilli, and were catalase-positive and oxidase-negative were considered as *Corynebacterium* spp. Bacteria growing on SBA and MAC, Gram-negative rods, oxidase-negative and catalase-positive were identified as *Enterobacteriaceae*. For further characterization of *Enterobacteriaceae*, colonies were cultured on EMB. Colonies with a light pink zone of precipitation, indole positive, hydrogen sulfide negative and citrate negative, and produced metallic shine on the surface of EMB were identified as *E. coli*. Pink mucoid colonies, indole-negative, citrate-positive, and nonmotile bacteria were identified as *Klebsiella* spp., while motile bacteria were classified as *Enterobacter* spp. Swarming and foul odor in blood agar, pale colonies on MAC, alkaline (red) slant and acidic (yellow) but with hydrogen sulfide production in TSI were identified as *Proteus* spp.

### Description of variables

#### Clinical mastitis

A cow has CM when milk from one or more quarters shows abnormal color, viscosity or consistency in addition to inflammatory signs of the udder. CM cases that occurred 21 days after a previous case in the same or a different quarter were considered as new incidents (recurrence)^12^.

#### Teat end condition (teat end score)

The teat end condition was scored according to Mein et al.^22^ on a scale of 0 – 4. According to this scoring system; normal teat and no deformation (0), the teat orifice is slightly more open, appears rougher, and has lost its circular appearance (small hyperkeratinized/rough teat end condition) (1), some small roughness appears in the form of keratin fronds, protruding up to 2 mm from the raised teat orifice (moderate hyper keratinized teat end condition) (2), a very rough orifice, with keratin protruding all around the teat sphincter (severe hyper keratinized teat end condition) (3); and a rough keratin protrusion of up to 4 mm, with the sphincter giving the impression of having been turned inside out (very severe hyper keratinized) (4).

#### Udder and leg hygiene of cows (cow dirtiness)

The udder and leg hygiene (ULH) of each cow was assessed and scored based on a four-point scale (1–4) as described by Schreiner and Ruegg^23^ as follows: ULHS of '1' meant no contamination of the skin of the rear of the udder or the hind limb between the hock and coronary band; ULHS '2' slightly dirty (2–10% of the area covered in dirt); ULHS '3' moderately dirty (10–30% of the area covered in dirt); and ULHS '4' caked-on dirt (> 30% of these areas completely covered in dirt).

### Statistical analysis

Data from the questionnaire survey, follow-up study, and laboratory analysis of milk samples were registered, filtered, and coded in MS Excel spreadsheets. All statistical procedures were performed using STATA Version 14 for Windows (Stata Corp, College Station, Texas). The incidence rate of CM (IRCM) at the animal level was calculated as the number of animals that had a new case of CM divided by the total animal time at risk, and the results were scaled into 100 cow-years^20^. Kaplan–Meier (K–M) life table analysis was used to compute and describe the cumulative survival probability and cumulative incidence of CM in tabular form. Moreover, the K–M curve was used to plot the cumulative survival data to the lactation stage. The log-rank test was used to test the hypothesis that there is no difference in the survival curves between the levels of categorical variables and to select potential variables for multivariable analysis of risk factors. The association of various potential risk factors with the IRCM was tested using a multivariable Cox proportional hazards regression model. Only variables with a p-value < 0.25 in the log-rank test were subjected to multivariable Cox regression analysis after checking for multicollinearity using Kruskal gamma statistics. The final model was built by stepwise backward elimination of variables that were not significant at the 5% level. At each step during model building, control of potential confounders was carried out. A variable was considered a confounder if the coefficients of the remaining variables changed by 20%, and these were retained in the model even if they were not significant. The final model was tested for the proportional hazards assumption by using the Schoenfeld and the scaled Schoenfeld residuals (phtest)^24^. If the tests in the table are not significant (p > 0.05), we cannot reject proportionality and assume that there is no violation of the assumption of proportionality. *P* values < 0.05 in the final model were considered to be statistically significant.

### Eithical statement

The study was ethically approved by the Institutional Research Ethics Review Committee of Hawassa University. All methods were performed following the relevant guidelines and regulations. Before conducting the study, the objectives, expected outcomes, and benefits of the study and that the study would not cause any harm or risk to the animals were explained to the dairy farm owners or managers who participated in the study. Written informed consent was obtained from all 19 dairy farms.

## Results

### General information and management practices on dairy farms

The majority (79%) of the farm owners or managers had secondary to tertiary education. In 84.2% of dairy farms, managers are employed, while only 15.8% are run by owners. Only three farms (15.8%) had an employed veterinarian who monitored the health of the cows. All dairy farms (94.7%), with one exception, practice tie-stalls, and zero-grazing systems. The average milk production per cow on the farms was 14.7 Lt per day (Range: 4–34 Lt). Post-milking teat dipping with chemicals (tincture of iodine) was practiced by only one farm (5.3%). More than half (57.9%) of the dairy farms had poor sanitary facilities during the observation period. The ventilation system was poor in 68.4% of the dairy farms, and 63.2% of the farms did not use bedding material. About 57.9% of the dairy farms washed the entire udder and dried it with a towel before milking the cows, and 42.1% only washed the teats and did not use towels. Dry cow therapy was not practiced on any of the farms; however, chronically infected cows were culled on 68.4% of the farms. About 84.2% of dairy farms reported clinical mastitis as a problem in their cows, and to prevent spread to other cows, they milked cows with mastitis last after milking healthy cows. Additionally, 89.5% of the farms stated that they used antimicrobials to treat CM cases. However, in at least 84.2% of farms, farm owners, employees, or managers of the farms typically prescribed and administered antibiotic treatments for CM cases, usually by intramuscular injection, whenever they occurred. The most commonly used antimicrobials were oxytetracycline or a penicillin–streptomycin combination. The discarding of milk after antibiotic treatment was dependent on the severity of the CM. Milk was not discarded when cases were less severe. Approximately 78.9% of the farms discarded milk during antimicrobial treatment, but only from the affected quarters and until clinical symptoms resolved. Only 2 farms (10.5%) complied with the milk withdrawal period according to the manufacturer’s instructions. The other 21.1% did not discard milk but fed it to the calves for 3–4 days. In addition, one of the farms uses commercially available disinfectants in barns, particularly during the occurrence of multiple CM cases and rainy seasons, to reduce the incidence of cases. Another farm regularly uses ash after barn cleaning as a control and preventive strategy.

### Incidence rate of clinical mastitis

The 217 cows enrolled in the study were at risk for a total of 94.4 cow years. A total of 79 cows developed CM during the study period, resulting in an estimated overall IRCM of 83.72 cases per 100 cow-years (95% CI: 63.2 to 98.2), considering only the first case. At the farm level, the incidence rate varied from 0 to 222.22 cases per 100 cow years at risk. Cases of CM were recorded in all farms except one (Table 1). During the follow-up period, a total of 14 cows were withdrawn from the study (lost to follow-up) before developing CM due to sales (n = 13) and death (n = 1).

**Table 1:** Farm level and overall incidence rate of clinical mastitis in the dairy farms of Hawassa city.

Farm ID	No. cows at risk	Time at risk (weeks)	No. cases	Incidence rate/100 cow-weeks	Incidence rate/100 cow-years
H01	14	351	4	1.14	59.26
H02	8	164	4	2.44	126.83
H03	14	330	4	1.21	63.03
H04	23	318	15	4.72	245.28
H05	5	134	2	1.49	77.61
H06	14	331	5	1.51	78.55
H07	17	324	6	1.85	96.30
H08	6	139	2	1.44	74.82
H09	29	671	9	1.34	69.75
H10	7	171	4	2.34	121.64
H11	6	154	1	0.65	33.77
H12	6	114	4	3.51	182.46
H13	8	109	2	1.83	95.41
H14	8	117	5	4.27	222.22
H15	13	396	3	0.76	39.39
H16	7	170	3	1.76	91.76
H17	15	372	5	1.34	69.89
H18	4	156	0	0.00	0.00
H19	13	386	1	0.26	13.47
Overall	217	4907	79	1.61	83.72

### Reoccurrence of clinical mastitis and non-functional teats

Of the 79 cows that developed CM, 18 (22.8%) cows had multiple cases of CM in the same quarter or different quarters. Reoccurrence was higher in HF local crosses (94.4%) than Jersey-local crosses (5.6%) and in multiparous cows (77.8%) than in primiparous cows (22.2%). Of the 79 cows that developed CM, 10 cows (12.65%) had at least one non-functional teat that had stopped milk secretion. Non-functioning teats were found more frequently in the hindquarters (70%) than in the forequarters (30%). Non-functional teats were observed more often in multiparous cows than in uniparous cows. All the non-functional teats were observed only in Holstein–Friesian local crossbred cows.

### Kaplan‒Meier survival analysis

**Figure 1:**
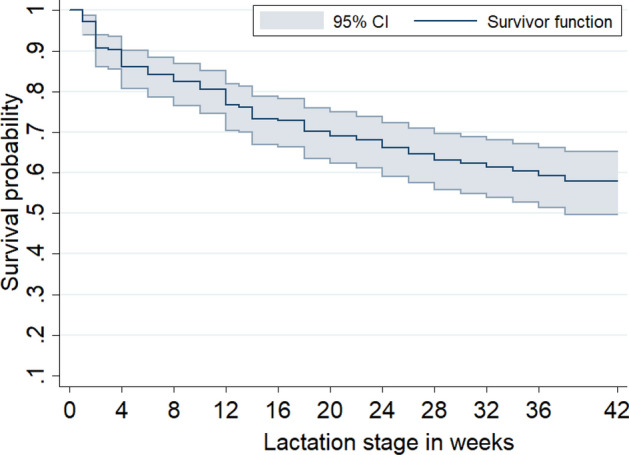
Kaplan‒Meier survival curve of CM in cows from calving to the end of lactation.

The cumulative survival probability of cows against CM by lactation length is shown in Fig. 1. As seen from the graph, there is a steady decrease in the survival probability of cows with increasing lactation length. From the K–M life table analysis (Supplementary file 2), the cumulative probability of a cow surviving or remaining free of CM at the end of lactation or follow-up period was determined to be 57% (95% CI: 48.6%—64.5%). In other words, the present study showed that the cumulative incidence of CM, or the probability that a cow would develop CM during lactation, was 43% (95% CI: 35.5–51.4%). Furthermore, the K–M life table analysis showed that the incidence rate is higher in the first four weeks of lactation and then decreases with increasing lactation stage.

### Mastitis risk factors

#### Univariable analysis

In the univariable analysis, 17 potential risk factors described in the data analysis section were tested by the K–M method and log-rank test for their effect on the incidence of clinical mastitis and to select potential candidates for the multivariable analysis. Of the factors examined, breed, parity, teat end condition, mastitis history, udder and leg hygiene score, barn hygiene, teat end shape, udder and teat preparation before milking, and culling of chronically affected cows had a p-value < 0.25 and were selected as potential candidates for multivariable Cox regression analysis (Table 2). The distribution of data across levels of the variables considered in the multivariable analysis is given in Table 3.

**Table 2:** Results of the log-rank test of the survival curves for each group of categorical variables.

Variables	$\chi$^2^	Degree of freedom	p-value
Age (≤ 5/ > 5 yr)	1.20	1	0.2728
Breed (Jersey/HFriesian)	1.97	1	0.1602
Parity (primiparous/multiparous))	5.8	1	0.0160
Average milk yield (≤ 10/ > 10 lit)	0.59	1	0.4406
Mastitis history (yes/no)	16.07	1	0.0001
Teat end shape (pointed/flat/round)	4.73	2	0.0939
Teat end condition (0–4)	44.06	4	0.0000
ULHS (1–4)	9.75	3	0.0208
Educational status (reading and writing/secondary/tertiary)	0.93	2	0.6285
Barn hygiene (good/poor)	8.42	1	0.0037
Ventilation (good/poor)	0.50	1	0.4782
Herd size (< 50/ ≥ 50)	1.30	1	0.2535
Use of towel (yes/no)	0.18	1	0.6681
Preparation of udder and teats (whole udder/teats only)	2.60	1	0.1072
Culling chronically affected cows (yes/no)	1.62	1	0.2032
Bedding (yes/no)	1.32	1	0.2505
Veterinarian on the farm (yes/no)	0.46	1	0.4986

**Table 3:** Distribution of data across levels of variables used in the Cox regression model.

Risk factor	Category	No cows at risk	Time at risk (cow years)	No cases	Incidence rate/100 cow years	95% CI for IR	
						Lower limit	Upper limit
Breed	Jersey cross	26	14.31	7	48.9	23.3	102.6
	HF crosses	191	80.12	72	89.9	71.3	113.2
Teat end shape	Pointed	117	53.5	39	72.9	53.3	99.8
	Round	75	28.23	33	116.9	83.1	164.4
	Flat	25	12.7	7	55.1	26.3	115.6
ULHS	1	73	32.13	25	77.8	52.6	115.2
	2	64	31.71	18	56.8	35.8	90.1
	3	43	18.61	16	86	52.7	140.3
	4	37	11.98	20	166.9	107.7	258.8
Mastitis history	No	155	71.64	44	61.4	45.7	82.5
	Yes	62	22.79	35	153.6	110.3	213.9
Parity	Primiparous	58	26.52	13	48.9	28.4	84.3
	Multiparous	159	67.87	66	97.2	76.4	123.8
Teat end score	0	148	64.05	46	71.8	53.8	95.9
	1	37	18.99	13	68.5	39.8	117.9
	2	15	6.85	6	87.6	39.4	195
	3	12	4.17	9	215.8	112.3	414.8
	4	5	0.37	5	1351.4	562.5	3246.7
Barn hygiene	Good	113	53.33	31	58.1	40.9	82.7
	Poor	104	41.1	48	116.8	88.0	155.0

#### Multivariable analysis

After assessing multicollinearity, variables with a p-value less than 0.25 in the log-rank test (shown in Table 3) were subjected to the multivariable Cox regression analysis. In the final model, previous history of mastitis (p = 0.003), parity (p = 0.03), teat end condition (p < 0.001), and barn hygiene (p = 0.007) were found to be significantly associated with the incidence rate of CM, while breed, teat end shape, ULHS, and preparation of the udder and teats before milking were not significantly (p > 0.05) associated and were consequently excluded from the model. Accordingly, it was found that with the effects of other variables held constant, the risk of CM in cows with a history of mastitis and in multiparous cows was approximately twofold higher than in their counterparts. Cows with very severe keratinized teat ends had a 7.7 times higher risk of CM than cows with normal teat end conditions. It was also found that the risk of CM was 1.89 times higher in cows kept in poorly cleaned barns than in cows kept in relatively better hygienic conditions (Table 4). The final model was tested for the proportional hazards assumption and found not to violate the assumption (global test $\chi$^2^ = 0.98, df = 4, p = 0.9122) (Table 5). Therefore, parity, history of mastitis in the previous lactation, teat end condition, and barn hygiene remained potential risk factors for CM.

**Table 4:** Multivariable Cox regression model of risk factors associated with clinical mastitis at Hawassa dairy farms.

Risk factor	Category	HR	SE	z	p	95% CI for HR
Mastitis history	No	Ref				
	Yes	2.04	0.5	2.95	0.003	1.27–3.27
Parity	Primiparous	Ref				
	Multiparous	1.96	0.6	2.17	0.030	1.07–3.61
Teat end score	0	Ref				
	1	0.88	0.3	-0.40	0.690	0.48–1.64
	2	1.19	0.5	0.39	0.694	0.50–2.83
	3	1.69	0.7	1.36	0.175	0.79–3.62
	4	7.72	3.9	4.05	0.000	2.87–20.76
Barn hygiene	Good	Ref				
	Poor	1.89	0.4	2.69	0.007	1.19–3.0

**Table 5:** Test of proportional hazards assumption.

Variable	rho	$\chi$^2^	df	p
Mastitis history	0.00816	0.01	1	0.9385
Teat end score	−0.04660	0.23	1	0.6290
Parity	−0.05502	0.24	1	0.6247
Barn hygiene	0.07441	0.45	1	0.5034
Global test		0.98	4	0.9122

### Bacteria isolation

Milk samples were collected from 57 selected cows affected by clinical mastitis and cultured for bacterial isolation. Of these, bacterial growth was observed in 52 (91.2%) samples while no growth was observed in 5 (8.8%). Single bacterial growth was found in 45 (86.5%) of the positive samples, while mixed bacterial growth was observed in 7 (13.4%) samples. A total of 59 isolates of bacteria belonging to eight genera were detected in the positive cultures. At the bacterial species level, *S. aureus* (28.1%) was the predominant species isolated followed by *E. coli* (21.1%) (Table 6).

**Table 6:** Bacterial species isolated from mastitis-positive cows (N = 57).

Bacteria Isolates	No of samples	Prevalence (%)
*Staphylococcus aureus*	16	28.1
NAS	3	5.3
CNS	7	12.3
*E.coli*	12	21.1
*Bacillus* spp*.*	8	14.0
*Streptococcus* spp*.*	8	14.0
*Enterobacter* spp*.*	2	3.5
*Klebsiella* spp*.*	1	1.8
*Corynebacterium* spp*.*	1	1.8
*Proteus* spp*.*	1	1.8
No growth	5	8.8

## Discussion

This is one of the few longitudinal studies in Ethiopia that was designed to estimate the incidence rate of CM in dairy cows, identify the associated risk factors and isolate bacterial causes on dairy farms of Hawassa City in southern Ethiopia. The incidence rate of CM in the present study was 83.7 cases per 100 cow-years at risk, and the cumulative incidence was 43%. Studies on the incidence rate of CM are very few in Ethiopia, so it is difficult to compare and discuss the present findings in the context of our country. Nonetheless, the current incidence rate is much higher than a previous finding of 21.26 cases per 100 cow years at risk in Gondar town in northern Ethiopia^25^. The higher incidence rate in the present study may be related to a longer observation period of the cows and a larger sample size compared to a six-month follow-up by Almaw and his co-workers. The current finding is also considerably high when compared to incidence rates reported from other countries, such as 43.3 cases per 100 cow-years on smallholder dairy farms in Tanzania^26^, 13 to 40 cases/100 cow years in different countries, and housing types in Europe^5^, 23 cases/100 cows in Canada^10^, 43.9 cases per 100 cow-years in Bangladesh^27^. These variations in incidence rates may be related to differences in geographical location, management practices, and breed of cows.

This study found a significant (p = 0.03) association between the incidence rate of CM and parity. Holding the effect of other factors constant, the risk of CM in multiparous cows was 1.96 times higher than that in primiparous cows. A similar finding was also reported by other studies in different countries^8,28,29^. An increased risk of CM in multiparous cows could be attributed to sphincter relaxation and teat canal patency in older cows. Moreover, the median ligaments that support the teat also relax with age, causing the udder to sag, making it more prone to mastitis^29^.

The risk of CM in cows with a history of mastitis in previous lactation was twice as high as in cows without such a history, other factors being held constant (p = 0.003). This finding is consistent with other studies showing that the presence of CM in the previous lactation period increased the risk of CM by three to four times^11,28,30^. Furthermore, a study by Olivera et al.^28^ in Brazil shows that the presence of mastitis in the previous lactation increases the risk of CM by 20-fold in multiparous and 18-fold in heifers. It is well established that CM is a recurring event that leads to a higher susceptibility to further episodes of mastitis in the same or the next lactation^6,7^. It is also hypothesized that the higher risk of CM in cows with a history of mastitis in the present study may be due to the inappropriate use of antimicrobials to treat cases of mastitis.

The condition of the teat ends throughout a cow's life is useful information for the mastitis control program. The results of this study showed that cows with very severe keratin frond growth (teat end score 4) had a 7.72-fold higher risk of developing CM than cows with normal teat ends (teat end score 0) (p < 0.001). Consistent with the present study, studies in many other countries have also shown that poor teat end condition is a risk factor for CM^8,11,31,32^. The teat end or orifice is an important first line of defense to protect an udder from invading mastitis pathogens^33^. Therefore, the higher incidence of CM in cows with hyperkeratinized teat end conditions could be related to high contamination of the teat ends by bacteria and infiltration of microorganisms into the glandular tissue due to damage to the teat opening or teat canal. Bhutto and co-workers^31^ concluded that the physical condition of bovine teats is an indicator of the quality of the environment, milking management, and milking system used on a dairy herd and it can be used as an indicator of the risk of intramammary infections.

This study has further shown that the incidence rate of CM is significantly (p = 0.007) related to barn hygiene. It was found that cows kept in poorly sanitized barns had a 1.89 times greater risk of developing CM than cows that were kept in relatively better hygienic conditions. We observed that poor barn hygiene resulted in soiled udders and hind legs, which ultimately led to an increased incidence of CM. The study showed that of the 19 farms examined, 11 (58%) had unsanitary stalls, and 75% of the cows on these farms had slightly to heavily soiled udders and hind legs (scoring 2–4). We also found that there was a significant association between the incidence rate of CM and udder and leg hygiene scores, although this was not evident in the multivariable analysis due to the confounding effect of barn hygiene. In agreement with the present study, Neja et al.^35^ from Poland reported that higher udder contamination caused a significant difference in the development of mastitis. The authors further stated that increased soiling of the udder was influenced by the hygiene of the cow's environment and the facilities in the cowshed.

A very frustrating aspect of CM is its recurring nature^5^. In this study, it was observed that 22.8% of cows with CM, experienced additional CM episodes in the same or another udder quarter during the same lactation. This finding is consistent with other studies elsewhere^5,36,37^. According to the studies, the most important risk factors for a CM recurrence are parity (the risk is greater in older cows), higher milk production, which pathogen species were involved in the preceding case, and whether antibiotic therapy was strictly adhered to previously. Consistent with this, we found that the rate of recurrence was considerably higher in multiparous cows (76.5%) than in primiparous cows (23.5%) and in cows producing more than an average of 10 L of milk per day (88.9%) than in cows producing less than 10 L (10.1%). The reason for recurrent mastitis can be either a persistent infection of the bovine mammary gland by a mastitis pathogen due to treatment failure or reinfection of a quarter or udder after bacteriological cure^5,36,38,39^. We hypothesized that the recurrence of CM in the current study could be attributed to treatment failure since the prescription and administration of antimicrobials for cases were performed by farmers/managers themselves who had no veterinary knowledge, and the route is only parenteral. Therefore, in addition to failure to administer intramammary infusions, failure to select the appropriate antimicrobials and non-adherence to the treatment regimens are to be expected. Furthermore, it should be noted that this indiscriminate use of antimicrobials can lead to the emergence of a drug-resistant strain of bacteria and pose a risk to public health.

This study found that a variety of contagious and environmental bacteria cause CM in dairy cows. *Staphylococcus aureus* was the most common bacterium isolated from 28.1% of the cultured mastitis-positive milk samples, followed by *E. coli* (21.1%). The spectrum of bacteria dominated by *S. aureus* identified in the present study is consistent with those identified by a previous study on clinical mastitis in Ethiopia^40^. Outside Ethiopia, studies conducted in Canada^10^ and India^41^ have also reported the predominance of *S. aureus* in clinical mastitis cases. However, in contrast to our study, the predominance of environmental bacteria has been reported from other countries such as the UK^11^, Bangladesh^27^, Brazil^28^, USA^42^, and China^43^. The predominance of *S. aureus* in the study area could be related to milking hygiene practices on the farm. It was found that milking was done by hand on all farms without exception and that milkers only wash their hands before milking the first cow, but not between milkings. Additionally, to wash other cows, they simply dip their hands in the water left over from washing previous cows. Thus, this practice creates a suitable condition for the easy transmission of bacteria from infected to unaffected cows through the milker’s hands or the use of contaminated water. It is well known that contagious bacteria such as *S. aureus* can be found on the udder or teat surface of infected cows and are the primary source of infection between infected and uninfected udder quarters or cows during milking^44^. Furthermore, the higher incidence of *S. aureus* in this study could also be related to the lack of post-milking teat dipping practices or dry cow therapy in almost all farms studied. Notably, the prevention of new infections depends on the practice of hygienic procedures. Separate and last milking of infected cows and dry cow therapy is more effective in eliminating infections than lactating treatment^45^. The occurrence of *E. coli* and other environmental microorganisms was most likely due to the contamination of cows’ udders and teats with manure, bedding, feedstuffs, dust, dirt, mud, or other materials in the cowsheds. Heavy soiling of barns increases bacterial growth and thus teats exposure to bacteria. As noted in the study the incidence rate of CM was significantly higher in cows kept in poorly sanitized barns.

### Limitations of the study

Cows monitored for clinical mastitis were recruited only from relatively large farms (10 or more cows) to increase the likelihood of getting lactating cows for follow-up. Farms with fewer than 10 cows were not considered and therefore, the study lacks information on how the management practices on these farms might influence the development of clinical mastitis in cows. The other limitation is that this study did not attempt to isolate the bacterial pathogens from recurrent CM cases, which would help determine which bacteria are important in the recurrence of the disease. We have only relied on bacterial culture to isolate bacteria from CM cases and due to the lack of species-specific primers for molecular analysis, we have been unable to identify bacteria at the species level, except *S. aureus* and *E.coli*. This is another limitation of the current study that needs to be addressed in future studies on CM.

## Conclusion

The current study found a higher incidence rate of clinical mastitis in dairy farms in Hawassa City. This suggests that the disease is spreading rapidly in dairy farms and requires due attention. The rapid spread of the disease could have a major impact on the production cycle of cows in general and the quantity and quality of milk production in particular and increase the use of antimicrobials. All this leads to negative effects on the economy of dairy producers. In addition, the high incidence of clinical mastitis increases discomfort in animals due to pain from the disease. In addition, a range of bacteria involved, the increased use of antimicrobials to treat the disease by non-professionals, and the absence of discarding milk from cows with mastitis or during antibiotic treatment by some dairy farms suggest a higher public health risk from the consumption of raw milk and its products. The study also found that multiparous cows with a history of mastitis in the previous lactation, who had severely keratinized teat ends and were housed in poorly sanitized barns were at a higher risk of developing clinical mastitis. The study further demonstrated that both contagious and environmental pathogens are implicated in causing clinical mastitis on dairy farms. Adequate awareness raising among dairy farmers is therefore required to enable them to implement routine mastitis prevention management practices, such as identifying and treating clinical cases; post-milking teat disinfection, proper hygiene of the cow barns and milkers, and the use of dry cow therapy.

## Supplementary Information

Supplementary Information 1.

Supplementary Information 2.

## Data Availability

All data generated or analyzed during this study will be provided by the corresponding author upon reasonable request.

## Abbreviations

CM: Clinical mastitis
CI: Confidence interval
HR: Hazard ratio
K–M: Kaplan–Meier
Ref: Reference
NAS: Non aureus staphylococci
CNS: Coagulase negative staphylococci
