# Supplementary material for: Incidence rate, risk factors, and bacterial causes of clinical mastitis on dairy farms in Hawassa City, southern Ethiopia
Source: Sci Rep. 2023 Jul 6;13:10945. doi: 10.1038/s41598-023-37328-1 (PMC10326075; doi:10.1038/s41598-023-37328-1)
Supplement: Supplementary file 1 — Supplementary Information 1. [file 41598_2023_37328_MOESM1_ESM.pdf]

Supplementary File 1: Map of Ethiopia showing the study area: Hawassa City

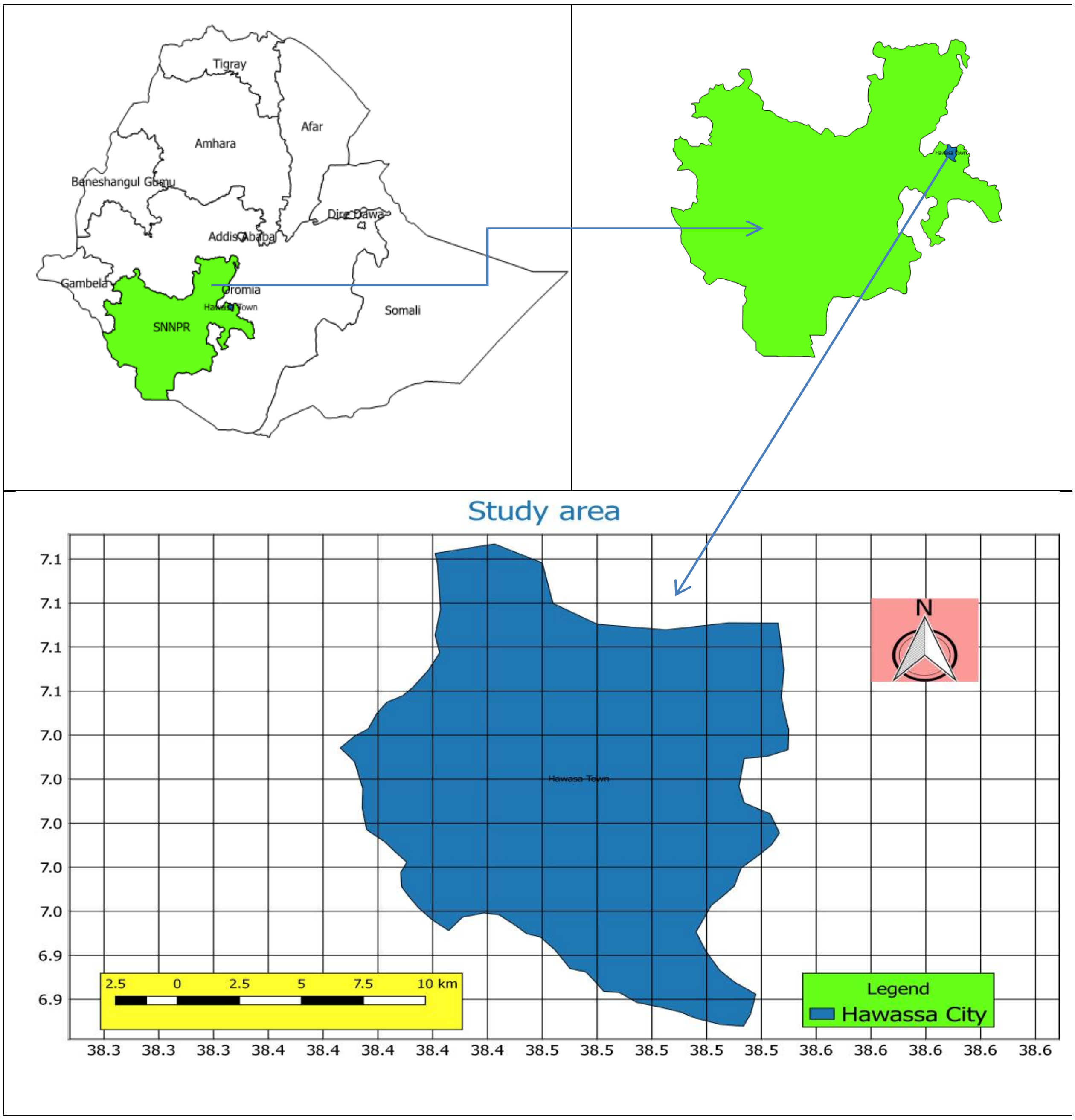

The map was created by the 2nd author of the manuscript using QGIS version 3.4.3 software: available at https://download.qgis.org/downloads/qgis-3.4.3.tar.bz2
