# Supplementary material for: Incidence rate, risk factors, and bacterial causes of clinical mastitis on dairy farms in Hawassa City, southern Ethiopia
Source: Sci Rep. 2023 Jul 6;13:10945. doi: 10.1038/s41598-023-37328-1 (PMC10326075; doi:10.1038/s41598-023-37328-1)
Supplement: Supplementary file 2 — Supplementary Information 2. [file 41598_2023_37328_MOESM2_ESM.pdf]

Supplementary File 2. The result of the K-M life table analysis shows the cumulative survival probability and cumulative incidence CM in cows throughout the lactation period

Lactation interval (weeks)	Cows at risk	Cases	Censored	Survival prob.	Std. Error	95% CI	Cum incidence
0-2	217	6	2	0.9722	0.0112	0.9392, 0.9874	0.0278
2-4	209	15	0	0.9024	0.0202	0.8543, 0.9352	0.0976
4-6	194	9	0	0.8606	0.0236	0.8067, 0.9004	0.1394
6-8	185	4	0	0.8420	0.0249	0.7860, 0.8844	0.1580
8-10	181	4	1	0.8233	0.0260	0.7654, 0.8681	0.1767
10-12	176	4	2	0.8045	0.0271	0.7449, 0.8516	0.1955
12-14	170	9	2	0.7617	0.0291	0.6986, 0.8133	0.2383
14-16	159	6	6	0.7324	0.0304	0.6674, 0.7867	0.2676
16-18	147	1	7	0.7273	0.0306	0.6619, 0.7821	0.2727
18-20	139	5	6	0.7005	0.0317	0.6333, 0.7578	0.2995
20-22	128	2	5	0.6894	0.0322	0.6214, 0.7476	0.3106
22-24	121	2	9	0.6775	0.0327	0.6087, 0.7369	0.3225
24-26	110	3	15	0.6577	0.0337	0.5872, 0.7191	0.3423
26-28	92	2	7	0.6428	0.0345	0.5708, 0.7060	0.3572
28-30	83	2	5	0.6269	0.0355	0.5531, 0.6919	0.3731
30-32	76	1	6	0.6182	0.0360	0.5435, 0.6844	0.3817
32-34	69	1	6	0.6089	0.0367	0.5330, 0.6763	0.3911
34-36	62	1	11	0.5981	0.0376	0.5205, 0.6673	0.4019
36-38	50	1	5	0.5855	0.0388	0.5054, 0.6571	0.4145
38-40	44	1	13	0.5699	0.0408	0.4859, 0.6453	0.4301
40-42	30	0	8	0.5699	0.0408	0.4859, 0.6453	0.4301
42-44	22	0	22	0.5699	0.0408	0.4859, 0.6453	0.4301
